# Autonomy support for the academic goal pursuit and subjective well-being of students with disabilities

**DOI:** 10.1080/28324765.2023.2255040

**Published:** 2023-09-10

**Authors:** Élodie Audet, Pascale Dubois, Shelby Levine, Richard Koestner

**Affiliations:** Department of Psychology, McGill University, Montréal, Canada

**Keywords:** autonomy support, subjective well-being, psychological need satisfaction, academic goal progress, self-determination theory

## Abstract

Students with disabilities often face greater challenges flourishing in postsecondary academic settings and achieving academic goals than their peers. Over an academic semester, 234 university students with registered disabilities (75.60% female, *M*_*age*_ = 22.30) were recruited to participate in a three-wave, longitudinal study. The present research utilized a Self-Determination Theory framework to examine how perceiving autonomy support (i.e., listening, providing choices and options) from close others related to psychological need satisfaction (i.e., feelings of autonomy, competence, and relatedness), progress on academic goals, and subjective well-being. Specifically, the results suggest that autonomy support was significantly related to psychological need satisfaction, goal progress, and subjective well-being. Results also suggest the relation of autonomy support to subjective well-being was mediated by psychological need satisfaction and goal progress. The findings have broader implications regarding the academic success and well-being of students with disabilities and aid in understanding how close others can provide meaningful support despite the difficulties encountered. Practical ramifications and directions for future research are discussed.

1.

Students with disabilities (e.g., health, physical, or mental conditions) face greater challenges thriving in postsecondary academic settings and have less success in pursuing academic goals than their peers (Boney et al., 2019; Harrington et al., 2021). Only around 20% of students with disabilities who engage in post-secondary education successfully graduate (Grogan, 2015), and they experience significantly fewer education and employment opportunities (Hirano et al., 2018). Hence, improving the accessibility of postsecondary education for these students in particular is a leading concern in various countries (e.g., U.S. Department of Education & Office of Special Education and Rehabilitative Services, 2020; Government of Quebec, 2021) especially since the number of postsecondary students with disabilities has been increasing globally in recent years (World Health Organization, [WHO], 2022). For instance, the current research was conducted in Québec, Canada, where as many as 5.6% of university students report having a disability, with a 50% increase in the years 2016–2017 to 2020–2021 (Association Québécoise interuniversitaire des conseillers aux étudiants en situation de handicap, [AQICESH], 2022). Despite the growing need to support these students, they still face multiple barriers to access and secure services (Los Santos et al., 2019; Toutain, 2019; Yusof et al., 2020). Addressing these barriers is essential as receiving services has proven extremely helpful in supporting students with disabilities to persevere with their postsecondary studies (Newman et al., 2021).

Therefore, it is important to acquire a broader understanding of academic goal support and progress for students with disabilities specifically, as research suggests they achieve lower educational outcomes, have significantly lower rates of educational completion, and face extra barriers in academic environments than their peers (Francis et al., 2019; WHO, 2022). For instance, they face more challenges managing academic obstacles and completing assignments (McGregor et al., 2016), and report frequently procrastinating on tasks because they were indecisive as where to begin (Sayman, 2015). In addition, students with disabilities generally experience lower well-being (Canha et al., 2016; Green et al., 2005) and often feel more overwhelmed, depressed, anxious, and lonely than their peers (Sayman, 2015). Thus, understanding how close others and motivational concepts may support students with disabilities in enhancing their academic goals pursuit and well-being is vital. The current study integrated a Self-Determination Theory framework (SDT; Ryan & Deci, 2017; Ryan et al., 2021), which provides a multidimensional conceptualization of human functioning.

Specifically, SDT examines the autonomous (rather than controlled) inclinations in behaviors (Ryan & Deci, 2017). Being more autonomous means acting whole-heartly because it is important and meaningful (Ryan & Deci, 2017; Ryan et al., 2021). Thus, individuals experience their behaviors and thoughts as being truly self-endorsed and harmonious. In contrast, being more controlled means acting with a sense of internal or external pressure (i.e., because of feelings of guilt, anxiety, seeking to please others, etc.). Plentiful of research has shown the beneficial outcomes of acting with autonomy, such as increases in well-being, positive affect, life satisfaction, and goal progress (e.g., Audet et al., 2021, Holding et al., 2017; Koestner et al., 2008; Levine et al., 2021).

Regardless of the numerous studies depicting the advantages of acting autonomously (Ryan & Deci, 2017; Ryan et al., 2021), researching factors that may enhance this beneficial way of being in students with disabilities is still greatly lacking. Consequently, the present research focuses on autonomy support from close others and the specific role it might have had on students’ three basic psychological need satisfaction (i.e., autonomy, competence, and relatedness), goal progress, and subjective well-being. These concepts are defined in the following sections.

## 2. Autonomy support

In recent years, autonomy support has emerged as one of the most effective types of goal support (Koestner et al., 2012, 2019). Autonomy support is described as having choices and options, being listened to with empathy, and genuinely sensing that your feelings and perspectives are acknowledged (Legate et al., 2012; Levine et al., 2021; Ryan & Deci, 2017). Autonomy support enhances numerous positive outcomes such as well-being, personal goal progress, positive affect, happiness, and psychological need satisfaction (Audet et al., 2021; Ebersold et al., 2019, Froiland et al., 2019; Levine et al., 2020; Ryan et al., 2021).

Perceiving autonomy support from close others might be especially helpful for students with disabilities, as close others often help with routine tasks, acquiring new skills, and providing gentle encouragements (Boney et al., 2019; Harrington et al., 2021). Such support related to academic success, learning, and skills in students with disabilities (Boney et al., 2019; Harrington et al., 2021). Altogether, receiving this type of support not only helped in goal setting and boosted academic goal progress, but students with disabilities also disclosed feeling more emotionally supported, more comfortable sharing thoughts and worries, and reported an increase in well-being (Boney et al., 2019; Harrington et al., 2021).

As explained above, students with disabilities in academic contexts face multiple extra barriers and challenges compared to their peers (Dipeolu et al., 2015; Wong et al., 2015). Therefore, the current research hypothesized that autonomy support from close others would enhance psychological need satisfaction (i.e., autonomy, competence, and relatedness), academic goal progress, and subjective well-being in students with disabilities. In fact, SDT views feeling autonomous in one’s life as so fundamental to experiencing well-being that it is even considered a basic psychological need (Ryan & Deci, 2017).

## 3. Psychological need satisfaction

As such, SDT advances that individuals have three basic psychological needs (i.e., autonomy, competence, and relatedness), and that satisfaction of these needs is essential to experience healthy growth, well-being, and functioning (Ryan et al., 2021). Specifically, *autonomy* refers to feeling volitional in one’s behaviors, *competence* refers to feelings of mastery and effectiveness in one’s environment, and *relatedness* refers to feelings of closeness and care in close relationships (Ryan & Deci, 2017; Vansteenkiste et al., 2020). Prior studies have demonstrated that autonomous behaviors are fostered in supportive environments that nurture the satisfaction of these needs (Ryan & Deci, 2017).

For example, psychological need satisfaction is related to various well-being outcomes in students without disabilities, such as enhancing goal pursuit (Werner & Milyavskaya, 2018), subjective well-being (Hope et al., 2019), positive emotions (Holzer et al., 2021), life satisfaction (Chen et al., 2015), and even mediated various well-being outcomes (Ebersold et al., 2019 Putri & Muttaqin, 2022). In addition, recent work further suggests that psychological need satisfaction is related to the quality of life in students with disabilities (O’Shea et al., 2021). Still, much more research is needed on how these processes operate for these students specifically. Thhe current study hypothesized that psychological need satisfaction would relate to academic goal progress and subjective well-being.

## 4. Academic goals

Personal goals occupy a substantial place in our day-to-day lives and often guide our behaviors and actions. Goals are frequently represented as “mental representations” of ideal outcomes that individuals are committed to attaining, and they provide a vitalizing source of energy that supports optimal development (Heckhausen et al., 2019). Not surprisingly, goal progress is thus associated with increased well-being, life satisfaction, and fulfilling relationships (Fitzsimons & Finkel, 2018; Levine et al., 2020). Still, research on the goal pursuit of students with disabilities remains scarce and suggests they have more difficulties in achieving academic goals than their peers (Boney et al., 2019; Harrington et al., 2021). Given the additional challenges faced when pursuing academic goals (i.e., harder time managing academic tasks and completing assignments, barriers accessing and securing services, feeling overwhelmed, etc.), examining how SDT-related concepts interact to optimize goal progress and subjective well-being with these students distinctively is fundamental, especially considering the motivational antecedents of these two outcomes.

Hence, SDT highlights the association between autonomy support, psychological need satisfaction, goal progress, and well-being (Ryan & Deci, 2017), and researchers have often shown that the effects of autonomous environments on well-being outcomes are mediated by psychological need satisfaction and goal progress (Audet et al., 2021; Koestner et al., 2019; Sheldon & Kasser, 1998). Autonomy support has repeatedly been related to increases in psychological need satisfaction and goal progress, which further fuels well-being (Deci & Ryan, 2000; Gorin et al., 2014; Koestner et al., 2012, 2015; Powers et al., 2008). On the whole, it was thus hypothesized that psychological need satisfaction and goal progress would mediate the relation between autonomy support and subjective well-being. In other words, it was predicted that autonomy support would predict psychological need satisfaction and goal progress, which would lead to an increase in subjective well-being across the academic semester.

## 5. Present investigation

The general aim of the present study was to examine how autonomy support from close others related to the progress on personal academic goals participants had set for themselves and their subjective well-being. Three hypotheses were derived. First, it was hypothesized that autonomy support would relate to psychological need satisfaction, academic goal progress, and subjective well-being (hypothesis 1). Second, it was hypothesized that psychological need satisfaction would relate to academic goal progress and subjective well-being (hypothesis 2). Finally, it was hypothesized that psychological need satisfaction and goal progress may act as a mediator between autonomy support and subjective well-being (hypothesis 3).

## 6. Method

### 6.1. Participants and procedure

A longitudinal study with 234 students registered with the university’s disability service provider (Office for Student Accessibility), was conducted over the course of a 13-week Winter semester at a large public Canadian university. Students were recruited in collaboration with the provider, which offers numerous services including helping registered students with individualized support plans (e.g., exam accommodations, note-taking aid, assistive technology, etc.); specially designed workshops, webinars, and sessions led by specialists (e.g., planning with purpose, assisting in the writing of essays, interviewing tips, energizing methods, etc.); support programs (e.g., tutor services, learning services, etc.); and extra assistance from advisors. To recruit participants, the study was advertised by the provider through email distribution and online blurbs targeting registered students with disabilities. Sample characteristics are found in Table 1.

**Table 1.** Sample characteristics

Age—M (SD)	22.3 (4.85)
**Self-reported Gender**	
Female	75.6%
Male	15.4%
Self-definition	4.3%
Other	3.4%
Preferred not to answer	1.3%
**Ethnicity**	
White	71.4%
Asian	17.9%
Middle Eastern or North African	7.7%
Hispanic or Latinx	5.1%
First Nations, Métis, or Inuit	2.6%
Black	2.1%
**Self-reported Disability**	
Anxiety	*n* = 144
Depression	*n* = 100
Attention-deficit/hyperactivity disorder	*n* = 90
Other mental health difficulties	*n* = 57
Medical conditions	*n* = 39
Learning disability	*n* = 35
Mobility/orthopedic disability	*n* = 22
Traumatic or acquired brain injury	*n* = 20
Autism spectrum disorder	*n* = 16
Visual impairment	*n* = 11
Hearing impairment	*n* = 2
Language impairment	*n* = 2
Intellectual disability	*n* = 1
Speech impairment	*n* = 1
Preferred not to respond	*n* = 5

*Notes. N* = 234. This distribution may not depict a representative sample of students with disabilities.

Three questionnaires were sent to participants. The first was shared at the beginning of the semester (T1; January), the second in the middle of the semester (T2; March), with a retention rate of 83.3%, and the last at the end of the semester (T3; May), with a final retention rate of 77.4%. The surveys were distributed through the online survey software Qualtrics, and participants had three weeks to complete them. At each follow-up, participants were reminded of the two academic goals they had set for themselves at T1. Participants who had not yet answered or completed the survey received up to two weekly reminders. Financial compensation of $20 was offered to participants for their participation and the study was approved by the University Research and Ethics Board.

### 6.2. Transparency and openness

The datasets generated and analyzed in the current study are not publicly available due to the fact that they constitute an excerpt of research in progress but are available from the corresponding author on reasonable request.^1^,^2^

### 6.3. Measures

#### 6.3.1. Academic goals

As part of the study, participants were asked to indicate two academic goals they intended to pursue during the first survey administered at the beginning of the Winter semester (T1; January). Participants were first given a description of personal goals and examples of academic goals were provided, such as “Pass biology with a B or better”, “Develop a study plan for the semester”, or “Reduce procrastination on assignments”. Participants were then asked to reflect and write their own personal academic goals.

#### 6.3.2. Autonomy support

At the beginning of the Winter semester (T1; January), participants were reminded of their academic goals and were asked to name one close other who supported them in their goal pursuits. Some examples were given, such as a family member (e.g., sister) or a close friend.^3^ Perceived autonomy support was assessed with a commonly used scale to measure academic goal support (Holding et al., 2021; Koestner et al., 2012, 2019; Levine et al., 2021). Adaptations of the autonomy support scale have been widely employed for assessing different perceptions of autonomy support in specific circumstances (Deci et al., 2006; Legate et al., 2012; Ryan & Deci, 2017).

The predictive validity has been shown in numerous meta-analytic reviews, indicating significantly positive associations with well-being, positive behaviors, satisfaction of the three psychological needs, and negative associations with distress (Burgueño et al., 2020; Mossman et al., 2022; Simon & Salanga, 2021; Slemp et al., 2018). To demonstrate construct validity, scores have been correlated with measures from various supporters, such as parents, siblings, peers, teachers, coaches, friends, romantic partners, and healthcare providers (Burgueño et al., 2020; Mossman et al., 2022; Simon & Salanga, 2021; Slemp et al., 2018). Across these, correlations were invariant and not moderated by the source of support, country of the sample, ethnicity, gender, age, publication status, or the operationalization of autonomy support (Burgueño et al., 2020; Mossman et al., 2022; Slemp et al., 2018) and criterion validity showed acceptable to good internal consistency (Burgueño et al., 2020; Zimmermann et al., 2020), indicating it as a psychometrically valid measure.

Of interest to the current research, meta-analytic reviews (varying across educational levels) also examined the predictive validity between autonomy support and educational outcomes. These suggest significant positive associations between autonomy support and academic performance (Cor, 2008; Lochbaum & Jean-Noel, 2015; Okada, 2023), academic achievement (Okada, 2023), and positive emotions (Lochbaum & Jean-Noel, 2015).

Perceived autonomy support was assessed at the beginning of the semester, with three items, for example, “I feel that this person understands how I see things with my goals”. Each set of items had options scaling from 1 (*strongly disagree*) to 7 *(strongly agree*). In the current dataset, autonomy support had a reliability of α = .87.

#### 6.3.3. Psychological need satisfaction

The Balanced Measure of Psychological Needs scale (BPNS) is a commonly used, reliable, and validated scale (Campbell et al., 2015; Chen et al., 2015; Ryan & Deci, 2017; Sheldon & Hilpert, 2012; Van der Kaap-Deeder et al., 2020). The predictive validity of the BPNS has been shown in numerous systematic reviews and meta-analyses, suggesting that psychological need satisfaction has a great impact on prosocial behaviors, academic success, and other well-being outcomes (Girelli et al., 2019; Liga et al., 2020; Ng et al., 2012; Tang et al., 2020). Acceptable internal consistency, convergent validity, discriminant validity, dimensionality, measurement invariance, and criterion validity support the measure across demographics, ethnicity, gender, and age (Girelli et al., 2019; Olafsen et al., 2021; Rodrigues et al., 2019; Tang et al., 2020). Correlations with measures of psychological well-being provided evidence for criterion validity (Girelli et al., 2019; Olafsen et al., 2021), and the predictive validity suggests need satisfaction yielded unique associations in the prediction of mental well-being across various populations (Heissel et al., 2019; Liga et al., 2020).

The BPNS was assessed at the beginning of the Winter semester (T1; January) and in the middle of the semester (T2; March), using a 3-item shortened version. Originally, the BPNS is composed of nine items, with three subscales (one per need) of three items. Of those, only the three most face-valid item per need were preserved to keep the questionnaire as brief as possible. The decision to reduce the scale and uniquely keep the most face-valid items was made in collaboration with the advisors from the Office for Student Accessibility & Achievement and the registered students who assisted in the development of the surveys.^4^

The need for autonomy was assessed with the item “I was free to do things my own way”, the need for competence with the item “I was successfully completing difficult tasks and projects”, and the need for relatedness with the item “I felt a sense of contact with people who care for me, and whom I care for”. Each item was rated on a seven-point scale, with options ranging from 1 (*not true at all*) to 7 (*very true*). In the current dataset, psychological need satisfaction had a reliability of α = .50 at T1, and α = .50 at T2.

#### 6.3.4. Academic goal progress

Academic goal progress was assessed with a widely used scale for measuring goal progress over time (Holding et al., 2017; 2021; Hope et al., 2016; Koestner et al., 2012; Levine et al., 2020). Numerous meta-analytic reviews have shown the discriminant and criterion-related validity of this instrument in predicting academic achievements and even well-being outcomes (Huang, 2012; Klug & Maier, 2015; Koestner et al., 2002; Vowels & Carnelley, 2020). The validity of self-reported goal progress has also been corroborated with the use of objective assessments, such as endorsing a multi-informant approach (Koestner et al., 2012) or including psychophysiological indicators of stress reactivity (Holding et al., 2021). The adequacy and potential of person-centered analyses in the goal literature have been highlighted in a research synthesis (Wormington & Linnenbrink-Garcia, 2017).

Goal progress for the two academic goals was assessed at the end of the semester (T3; May), using the three following items (Koestner et al., 2012) “I have made a lot of progress toward this goal”, “I feel like I am on track with my goal plan”, and “I feel close to achieving this goal”. Participants answered on a seven-point scale that ranged from 1 (*strongly disagree*) to 7 (*strongly agree*). A mean score of all six items across the two goals was computed. In the current dataset, this scale had a reliability of α = .88.

#### 6.3.5. Subjective well-being

Subjective Well-Being (SWB) assesses “global life satisfaction—an evaluative judgment of one’s life as a whole” (Diener etal., 1985, p. 91) by primarily examining the cognitive (i.e., life satisfaction) and the frequency to which pleasant and unpleasant emotions are experienced. It has been extensively researched and discussed by Diener and colleagues and is an indispensable component of positive psychological health (Diener, 2013; Diener et al., 1985; Larsen & Diener, 1985; Pavot & Diener, 1993).

Numerous meta-analyses indicate that the measure shows adequate validity, reliability, factor invariance, cross-situational consistency, temporal stability, and sensitivity to change, and did not vary significantly as a function of sample characteristics (Busseri, 2018; Diener, 2013; Luhmann et al., 2012; Pavot & Diener, 1993). Convergent validity has been demonstrated with informant reports (Costa & McCrae, 1980; Lyubomirsky & Lepper, 1999; Pavot etal., 1991; Sandvik et al., 1993; Schneider & Schimmack, 2009), ratings based on clinical interviews and writing samples (Danner et al., 2001), and by analysis of memory bias for positive and negative events (Sandvik et al., 1993). Divergent validity has been shown by negatively correlating with measures of psychological distress or happiness (Arthaud-Day et al., 2005;Busseri, 2018; Diener, 2013; Pavot et al., 2018). The three constructs exhibit discriminant validity from each other, while still substantially loading on a latent SWB factor, supporting the generalizability of their associations and the robustness of this conceptualization (Arthaud-Day et al., 2005; Busseri, 2018). Related to the current research, a recent meta-analysis found that academic achievements do not necessarily result in higher SWB (Bücker et al., 2018).

Despite its widespread acceptance and use, the validity and utility of SWB has been challenged at several levels of analysis, ranging from its conceptual basis (e.g., Ryan & Deci, 2001; Ryff & Singer, 2008) to specific concerns about its conceptualization (Schimmack, 2008; Schwarz & Strack, 1999). These critiques were refuted, confirming the validity and utility of SWB (Pavot, 2013).

Life satisfaction, positive affect, and reversed negative affect measures were combined to report on SWB at the beginning of the semester (T1; January) and the end of the semester (T3; May). All components were standardized before calculating the mean. Life Satisfaction was assessed with five-items, such as “The conditions of my life are excellent” on a seven-point scale that ranged from 1 (*strongly disagree*) to 7 (*strongly agree*). In the current dataset, the scale had a reliability of α = .84 at T1, and a reliability of α = .88 at T3. Participants also completed the nine-item scale of affect (Emmons, 1992) which included four positive (e.g., joyful) and five negative (e.g., frustrated) items. All items were rated on a scale ranging from 1 (*not at all*) to 7 (*extremely*). The positive items had a reliability of α = .89 at T1, and a reliability of α = .92 at T3, whereas the negative items were reversed coded and had a reliability of α = .76 at T1, and a reliability of α = .87 at T3.

### 6.4. Analytic plan

Preliminary descriptive analyses were conducted to show the means, standard deviations, and correlations of the variables of interest. The hypotheses are tested in the main analyses section. First, to test hypothesis 1, separate hierarchical multiple regression analyses were conducted to test whether autonomy support at T1 related to psychological need satisfaction at T2; goal progress at T3; and subjective well-being at T3. Next, to test hypothesis 2, separate hierarchical multiple regression analyses were conducted to test whether psychological need satisfaction at T2 related to goal progress at T3; and subjective well-being at T3. An α value was set at .05 to determine significance, and all analyses controlled for baseline measures of the outcomes, except for goal progress, as there was no baseline for this variable. Finally, to test hypothesis 3, mediation analyses using the method outlined by Hayes (2018) were performed on the sequential indirect effects of psychological need satisfaction at T2 and goal progress at T3 to test whether these variables may act as a mediator between autonomy support at T1 and subjective well-being at T3. The Bootstrapping method and 5,000 resample (Hayes, 2018) to obtain a 95% confidence interval for the indirect effect were used. An α value was set at .01 to determine significance. All analyses were conducted with the SPSS statistics software (Version 29).

## 7. Results

### 7.1. Preliminary analyses

Data screening found the residuals and variables of interest to be normally distributed, making the variables suitable for regression analyses. Specifically, the assumptions of linearity, independence, no multicollinearity, normality, and homoscedasticity were met. The pattern of missing data was analyzed using Little’s MCAR Test in SPSS (Pituch & Stevens, 2016). The results were not significant, *p* = 1.00, suggesting only small deviations from the MCAR pattern. Therefore, all available data were used in the analysis, using Full Information Maximum Likelihood (FIML) estimation.

Paired *t*-tests suggest no changes in psychological need satisfaction, *t*(178) = .03, *p* = .980, or subjective well-being, *t*(178) = 1.02, *p* = .310, across the semester. Table 2 presents the means, standard deviations, and correlations among autonomy support, psychological need satisfaction, goal progress, and subjective well-being. The results of more precise regression analyses, while also controlling for baseline of the outcomes measures, are presented in the main analyses section.

**Table 2.** Means, standard deviations, and correlations among all measures

	M(SD)	1.	2.	3.	4.	5.	6.
1. Autonomy Support	5.75(1.26)	-	.204**	.188**	.299***	.196**	.286***
2. T1 Subjective Well-Being	3.40(.93)		-	.586***	.411***	.190**	.587***
3. T1 Psychological Need Satisfaction	4.47(1.05)			-	.467***	.271***	.442***
4. T2 Psychological Need Satisfaction	4.44(1.07)				-	.427***	.384***
5. Goal Progress	4.34(1.52)					-	.244**
6. T3 Subjective Well-Being	3.46(1.02)						-

Notes: ***p < .001, **p < .01.

### 7.2. Main analyses

Separate hierarchical multiple regression analyses were conducted on the variables of autonomy support, psychological need satisfaction, goal progress, and subjective well-being. All analyses controlled the baseline measures of the outcomes, with the exception of goal progress as mentioned above. The standardized regression coefficients, along with the *t*-values and the *F*-tests of the change in *R*^2^ are presented in Table 3 regarding autonomy support, and in Table 4 regarding need satisfaction.

**Table 3.** Hierarchical regressions of the effects of autonomy support from close others on psychological need satisfaction, goal progress, and subjective well-being

	Change in Psychological Need Satisfaction				Goal Progress				Change in Subjective Well-Being			
	*b*	*t*	*F testΔ*	*R*^*2*^ *Δ*	*b*	*t*	*F testΔ*	*R*^*2*^ *Δ*	*b*	*t*	*F testΔ*	*R*^*2*^ *Δ*
Baseline DV	.50***	7.38	*F*(1,162)=54.52	.25***	/				.58***	9.04	*F*(1, 163) = 81.71	.34***
Autonomy Support	.22**	3.11	*F*(2, 162) = 33.52	.04**	.20**	2.73	*F*(1, 188) = 7.49	.04**	.17**	2.67	*F*(2, 163) = 7.13	.03**

Notes: ***p < .001, **p < .01.

**Table 4.** Hierarchical regressions of the effects of psychological need satisfaction on subjective well-being and goal progress

	Goal Progress				Change in Subjective Well-Being			
	*b*	*t*	*F test Δ*	*R*^*2*^ *Δ*	*b*	*t*	*F test Δ*	*R*^*2*^ *Δ*
Baseline DV	/				.59***	9.59	*F*(1, 177) = 92.03	.20***
Psychological Need Satisfaction	.43***	6.28	*F*(1, 172) = 39.41	.19***	.45***	7.73	*F*(2, 177) = 59.67	.29***

Notes: ***p < .001, **p < .01.

#### 7.2.1. Autonomy support

First, hierarchical multiple regression analyses were conducted to examine whether autonomy support from close others at T1 related to need satisfaction at T2. The results suggest that while controlling for baseline psychological need satisfaction, autonomy support was significantly related to need satisfaction in the middle of the semester, *p* = .002. Then, it was examined whether autonomy support related to academic goal progress at T3. The results suggest that autonomy support was significantly related to goal progress at the end of the semester, *p* = .007. Finally, analyses were conducted to test whether autonomy support related to subjective well-being at T3. Following past research on the matter, the results suggest that autonomy support was also significantly related to subjective well-being at the end of the semester, *p* = .008.

#### 7.2.2. Psychological need satisfaction

Furthermore, analyses were conducted to examine whether psychological need satisfaction at T2 was related to goal progress and subjective well-being at T3. Results suggest that psychological need satisfaction in the middle of the semester was significantly related to both outcomes of goal progress, *p* < .001, and subjective well-being, *p* < .001, at the end of the semester. Accordingly, these findings suggest that psychological need satisfaction may be helpful for the academic goal pursuit and subjective well-being of students with disabilities.

### 7.3. Mediation analyses

**Figure 1.**
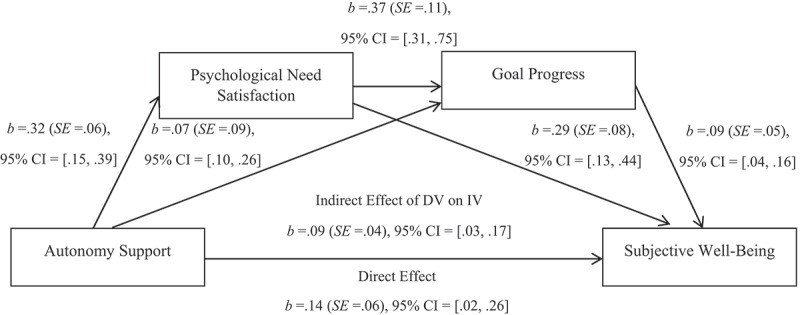
Direct and indirect effects of perceived autonomy support from close others on participants’ subjective well-being

A mediational analysis model (Figure 1) using the method outlined by Hayes (2018) was performed to test whether psychological need satisfaction at T2 and goal progress at T3 mediated the effects of autonomy support at T1 on subjective well-being at T3. First, results suggest that autonomy support was significantly related to psychological need satisfaction, *p* < .001, and goal progress, *p* = .002. Psychological need satisfaction was significantly related to goal progress, *p* = .012, and subjective well-being, *p* < .001. Similarly, goal progress was significantly related to subjective well-being, *p* = .013. Then, the indirect, direct, and total paths were estimated. The results suggest that the direct effect estimates were significant, *p* < .001, as were the total effect estimates, *p* < .001.

Finally, the Bootstrapping method and 5,000 resample (Hayes, 2018) to obtain a 95% confidence interval for the indirect effect were used. The results suggest autonomy support may indirectly effects changes on subjective well-being by relating to changes in psychological need satisfaction and goal progress, which may then lead to changes in subjective well-being. Since the intervals do not straddle 0, it supports the mediational model (Hayes, 2018) suggesting that autonomy support on subjective well-being may be mediated by psychological need satisfaction and goal progress.

## 8. Discussion

The general purpose of the current study was to examine how autonomy support from close others related to academic goal progress and subjective well-being of postsecondary students with disabilities. Following SDT research, the relation with satisfaction of the three psychological needs was also scrutinized. The results suggest that autonomy support at the beginning of the semester was related to psychological need satisfaction (i.e., autonomy, competence, and relatedness) in the middle of the semester, as well as goal progress and subjective well-being at the end of the semester. Furthermore, psychological need satisfaction and goal progress seemed to act as a mediator between autonomy support and subjective well-being. Taken together, these findings offer a new perspective on how close others may support students with disabilities and help them thrive during their academic endeavors.

### 8.1. Autonomy support and goal progress

First, as hypothesized, the results suggest that perceiving autonomy support from close others was related to progress on academic goals. Autonomy support is a personal interaction style in which the supporters attempt to take the perspective of the other and consider their thoughts, perspectives, and values (Ryan et al., 2021). Given that this type of support encourages the experience of volition, it follows that it likewise enhanced progress on academic goals. For instance, students may have felt understood and heard, which may have encouraged and motivated them to persist in their academic goals despite the difficulties encountered.

That is, multiple barriers impede students with disabilities from achieving their academic goals. For example, difficulties in social communication, self-advocacy, time management, and organization may interfere with the completion of course requirements (Burgstahler & Russon-Gleicher, 2015; Cai & Richdale, 2016). Also, the loss of structure that was once given before university, as well as the changes in routines, are often sources of distress among students with disabilities (Gelbar et al., 2014). The results, therefore, demonstrate that autonomy support, an interpersonal kind of support that feeds into the need to feel that actions truly emanate from the self (Ryan & Ryan, 2019), sustains goal progress in students with disabilities.

On the whole, these findings make sense as students with disabilities frequently report benefitting from having someone support their activities in choice-making, studying skills, and clarifying ambiguities when pursuing academic goals (Van Hees et al., 2015). Students with disabilities may thus find that receiving autonomy support, which includes behaviors such as genuinely being present for the other with a sense of non-judgment and curiosity (Roth et al., 2019), very helpful during their academic goal pursuits. In addition, it is important to consider the type of goals that were examined. While diverse personal goals have been largely studied, especially from an SDT perspective (Koestner et al., 2019; Levine et al., 2020), the current study examined academic goals specifically, which may be different from other types of goals.

### 8.2. Autonomy support and subjective well-being

Second, still in line with hypothesis 1, the results of the current study suggest that autonomy support from close others is related to subjective well-being at the end of the academic semester. Prior research has demonstrated that autonomy support enhances well-being, positive affect, happiness, healthy functioning, and mental health even during troublesome periods, such as stressful moments of the COVID-19 crisis or personal difficulties (Akram et al., 2022; Audet et al., 2021, Bülow et al., 2021; Levine et al., 2020, Neubauer et al., 2021; Ryan et al., 2021). The findings of the current study complement and extend these findings, by depicting that autonomy support also strengthens the well-being of students with disabilities.

Accordingly, close others, such as family members and friends, seem to hold meaningful roles in fostering well-being for students with disabilities despite the additional challenges they encounter in postsecondary academic settings (i.e., barriers accessing and securing services, facing more difficulties managing academic demands, etc.; De Los Santos et al., 2019; McGregor et al., 2016; Toutain, 2019; Yusof et al., 2020). Thus, researching what might magnify the subjective well-being of students with disabilities is essential given that they generally experience lower well-being and feel more depressed, anxious, overwhelmed, and lonely than their peers (Canha et al., 2016; Green, 2005; Sayman, 2015). Taken together, the current study suggests that perceiving autonomy support from close others did bolster the subjective well-being of students with disabilities at the end of the semester.

### 8.3. Psychological need satisfaction, goal progress, and subjective well-being

SDT puts forth the notion that individuals have three basic psychological needs (i.e., autonomy, competence, and relatedness) which are vital for individuals’ healthy development and growth (Ryan et al., 2021). As predicted in hypothesis 2, psychological need satisfaction in the middle of the semester was related to academic goal progress and subjective well-being at the end of the semester. These findings are coherent with prior research (DiMaggio et al., 2020, Koestner et al., 2019; Laporte et al., 2021; Vansteenkiste et al., 2020) and further suggest that it is valuable for students with disabilities to feel ownership over their experiences, socially connected with others, and sense they are efficiently capable of mastering their surroundings. When present, this ideal environment may have supported their academic thriving, both in terms of promoting academic goal progress and subjective well-being. These results reinforce the understanding that it is essential to foster autonomy-supportive environments for all students.

In addition, mediational analyses were conducted and suggested that psychological need satisfaction and goal progress may mediate the relation between autonomy support on subjective well-being. These findings are not too surprising as they have been shown in past research conducted with postsecondary students (Audet et al., 2021, Koestner et al., 2019; Laporte et al., 2021). Still, it is exciting to propose that environments that satisfy students’ psychological needs are likewise a place where students with disabilities can successfully pursue their academic goals and flourish.

## 9. Limitations

The current research has strengths, such as using a longitudinal design, validated measures, and a unique sample. Nevertheless, there are important limitations to be considered. Most importantly, the results are correlational and firm conclusions about causality cannot be inferred. Therefore, even though the hypotheses were drawn from past longitudinal and experimental studies (Koestner et al., 2020; Ryan et al., 2021), more research is needed to validate the findings. Equally noteworthy, participants reported a

participants reported a high rate of mental health disabilities, which does not represent the entirety of students with disabilities. It would be relevant for future studies to recruit a sample in which disabilities are more evenly distributed, as well as to conduct separate analyses for these groups. Relatedly, the current study included only students receiving services from the university’s disability service provider. As such, it would be interesting to consider how SDT variables differ between participants included in the study and those with similar disabilities who are not registered with universities’ disability service providers.

Next, the study was conducted with a university sample in which the majority was female and of European descent. Future research would benefit from including more heterogeneous samples to provide further insight, especially since there are points of intersectionality. For example, more work needs to be done in order to explore the intersection of those who are members of multiple marginalized groups, such as race, ethnicity, gender, gender expression, socio-economic status, sexual orientation, and more (Shpigelman et al., 2022; Smith et al., 2021). Forth, it may be possible that the correlations were due to confounding variables. For instance, other factors such as specific opportunities or constraints, differences in family and peer structures, or economic circumstances may have influenced the outcomes.

Fifth, the use of the brief scales and the rather low reliability of the psychological need satisfaction scale must be highlighted. While reducing the length of the survey to enhance students’ participation is an appropriate practice when conducting research with students with disabilities, they nevertheless are limited in their ability to fully capture the intricacies of the construct they are designed to assess. If time constraints and participant burden allow for it, future research could include more complex measures. Still, the results are consistent with the study hypotheses that SDT variables are truly useful for students with disabilities.

## 10. Implications and future directions

Nevertheless, the findings have valuable implications for practice and contribute to advancing the field of how to support students with disabilities despite the harshness of postsecondary academic settings. To illustrate, the general implications of the findings and how to effectively provide support for students with disabilities could be useful to the educational staff, school psychologists, counselors, and close others who accompany students with disabilities in postsecondary settings. That is, these individuals could benefit from learning how to provide autonomy support (e.g., listening, showing compassion, providing choices and options) and the benefits associated with taking this approach for students with disabilities (i.e., enhancing goal progress and subjective well-being).

In addition, this knowledge could benefit from being promoted in broader contexts. For example, new programs and services developed for students with disabilities could be implemented, with workshops explaining how to provide autonomy support or where students with disabilities could learn how to elicit it. This would enhance the knowledge of how close others can deliver the most optimal kind of support while simultaneously guiding students with disabilities on how to evoke this advantageous type of support from those around them. In sum, academic institutions and policymakers should recognize the indispensable role of autonomous environments and close others for postsecondary students with disabilities in achieving academic success and well-being.

Moreover, an important proportion of students with disabilities do not register with on-campus disability services, especially students with hidden disabilities (Newman & Madaus, 2015). Thus, cultivating autonomous environments for all students, whether they prefer to disclose their disabilities or not, is imperative both across campus and with close others. This is exceptionally true considering the substantial effects of the COVID-19 crisis, where students, with and without disabilities, are struggling now more than ever before (Chen et al., 2022; Dickinson et al., 2021; Lanza et al., 2022; Sandner et al., 2022; Theberath et al., 2022).

## 11. Conclusion

Despite the encouraging findings that students with disabilities are attending postsecondary education at higher rates, multiple obstacles continue to impede their progress and successful completion of academic goals (Boney et al., 2019; Francis et al., 2019; Harrington et al., 2021). The present research examined how perceiving autonomy support from close others related to psychological need satisfaction (i.e., autonomy, competence, and relatedness), progress on academic goals, and subjective well-being of students with disabilities. As hypothesized, the results suggest that over time, autonomy support from close others was related to psychological need satisfaction, goal progress, and subjective well-being. Furthermore, psychological need satisfaction and goal progress mediated the relation between autonomy support and subjective well-being. The results, therefore, support prior research demonstrating that this interpersonal kind of support also sustain the thriving of students with disabilities.

## Data Availability

The datasets generated and analyzed during the current study are not publicly available due the fact that they constitute an excerpt of research in progress but are available from the corresponding author on reasonable request.

## Notes

1. Measures and supplemental analyses are available on OSF: https://osf.io/etdgm/?view_only=f284e3e080fc494eaafbc8bf12775639

2. No other work has yet been published using this dataset.

3. Goal supporters had the following breakdown: 46.5% family members, 19.1% friends, 21.7% romantic partners, 4.8% counselor/therapist, and 7.9% other (i.e., coach, boss).

4. Thus, scales were accommodated to the population of interest and analyzed following prior SDT research (i.e., combining the three psychological needs (Ryan et al., 2021; Vansteenkiste et al., 2020)).
